# ‘Young crystallographers’ rejuvenate crystallography in Germany

**DOI:** 10.1107/S2056989024001695

**Published:** 2024-02-27

**Authors:** Florian Meurer, Jakob Möbs, Melanie Nentwich, Tina Weigel, Jan-Philipp Wöhrle

**Affiliations:** a Faculty for Chemistry and Pharmacy, University of Regensburg, Universitätsstr. 31, Regensburg, 93053, Germany; b Rossendorf Beamline, Helmholtz-Zentrum Dresden-Rossendorf, Dresden, Germany; c Department of Chemistry, University of Oxford, OX1 3PU, Oxford, UK; d Deutsches Elektronen-Synchrotron, DESY, Notkestr. 85, Hamburg, 22607, Germany; e Institute of Experimental Physics, TU Bergakademie Freiberg, Freiberg, Sachsen 09596, Germany; f Institut für Umwelt- und Naturwissenschaften, Universität Freiburg, Freiburg, Baden-Wurttemberg 79104, Germany; Universidad de Los Andes Mérida, Venezuela

**Keywords:** education, teaching, young scientists, crystallographic associations

## Abstract

Since its founding in 2013, the Young Crystallographers (YC) have become one of the most active working groups not only within their parent organization, the German Crystallographic Society (DGK), but also among other young crystallographers’ groups in Europe and the world. The aim of the YC is and always has been to support early-career researchers in the diverse fields of crystallography and the rejuvenation of the field on a national scale. Over the past decade, we have curated events, platforms, and educational content tailored to foster collaboration and knowledge transfer among young crystallographers. In this article, we introduce our group and show how this active and diverse community has shaped the rejuvenation of crystallography in Germany, strengthened by the support of our national society.

## Abstract 2

The Young Crystallographers were founded in 2013 within the German Crystallographic Association and have revitalized the field of crystallography by establishing collaboration and support among early career researchers, organizing educational events, and promoting scientific contributions on national and international stages.

## 1. Introduction and history

The year 2013 was a significant milestone for the German Crystallographic Society (DGK) as it was the year our working group, Young Crystallographers (YC), was established (Zschornak *et al.*, 2022 ▸). It was intended to be both an informative and interactive platform for young scientists and to promote continuous rejuvenation within the field. Like many other organizations, the DGK has to deal with declining membership figures and an aging community. The establishment of the working group was thus the first organizational step of the DGK to address those issues. We quickly became one of the most dynamic working groups, creating an active network of young researchers in the field of crystallography. However, the term ‘young’ does not refer to a specific age, but rather to the career stage at which scientific training is not yet completed, and the term ‘crystallographers’ includes researchers from any of the diverse scientific fields that use crystallographic methods.

The main tools of the working group have been from its inception:

**·** (bi)annual meetings, *e.g.*, symposia at the annual DGK conference and standalone meetings at industry partners and universities (see Section 2.1, Meetings),
**·** a blog with articles on crystallographic topics (see Section 2.2, *Blog articles*), and
**·** a monthly newsletter (see Section 2.3, *Communication: newsletter and social media*), informing members about upcoming conferences and courses, open job positions, as well as grants and awards.

Over the years, our various chairs and staff members improved this set of tools, adding new ones and replacing old ones, enhancing the outreach and impact of the working group. For instance, the originally established website forum became silent and was shut down. Instead, we created a profile on ResearchGate, which offers similar features on a more modern platform. Furthermore, in the years 2014 to 2017, we published a comprehensive list of recent publications by YC members. As a result of the significant effort required to maintain this list manually, changing data protection laws, and the rise of research network tools like ResearchGate, this tool was ultimately discontinued as well.

**Figure 1:**
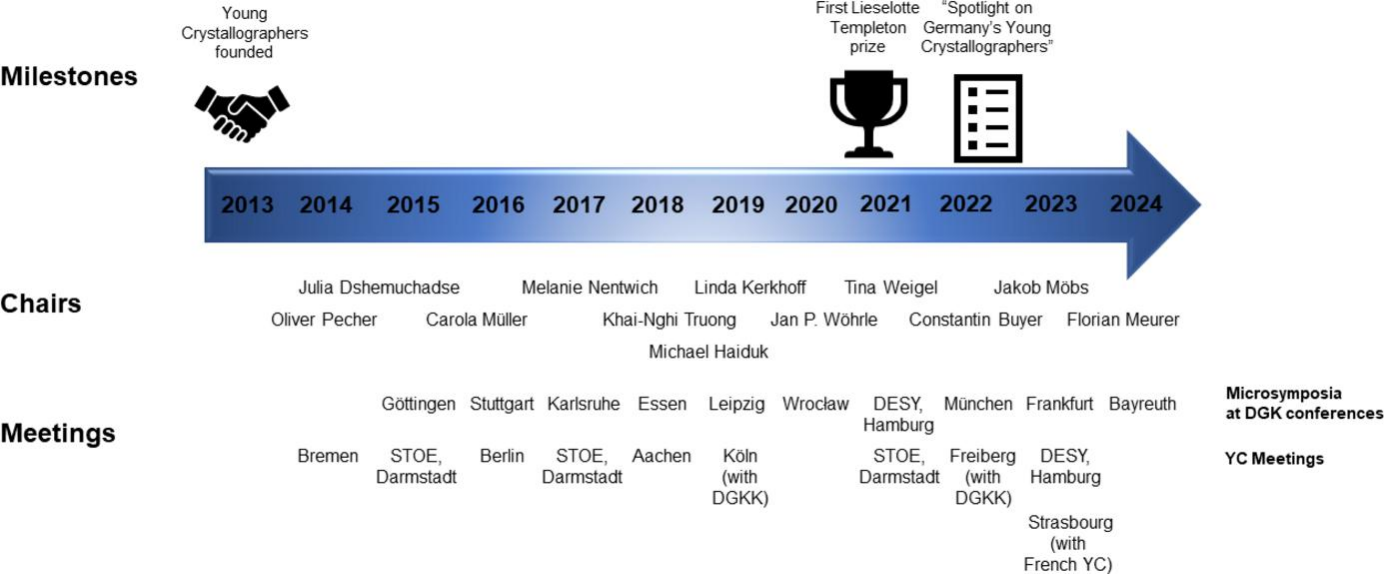
Timeline of the Young Crystallographers, including the respective chairs, major milestones, and conferences that were co-organized (DGK Microsymposia, hands-on lab meetings, meetings of the YC, and collaborative events with other associations such as the French Young Crystallographers and German Young Crystal Growers, DGKK).

In contrast, the annual meetings held every autumn at different universities and industry partners have been a complete success ever since the first one in 2014 (see Fig. 1 ▸ and Section 2.1, *Meetings*). As the 2020 conference could not take place due to the pandemic, we initiated a special issue of *Zeitschrift für Kristallographie* to offer potential participants an alternative platform for their scientific contributions (see Section 2.5, *Special issue, ‘Spotlight on Germany’s Young Crystallographers’*).

Additionally, we are a vocal presence on various social media platforms such as X (formerly Twitter), LinkedIn, Instagram, and Facebook. In 2021, the YC initiated a new prize dedicated to scientists at the earliest stages of their education: students. Each year, up to three prizes are now awarded for the best thesis from the previous year at Bachelor’s, Master’s, Diploma, or similar levels in the field of crystallography (see Section 2.4, *Lieselotte Templeton prize*).

Over the past ten years, eleven young scientists have gained experience and invaluable organizational and leadership skills by chairing the working group and taking over the responsibility of keeping the group active and thriving (see Fig. 1 ▸). Fortunately for the community, most former chairs decided to continue their support in various roles, *e.g.*, as secretary, treasurer, blog poster, or social media officer, even after the end of their official period of office. Furthermore, the chairs and their teams could always rely on their mentors Professor Ulli Englert (Aachen), Professor Dirk C. Meyer (Freiberg), and Professor Sibylle Gemming (Chemnitz) when advice was needed.

In addition to our efforts on behalf of young scientists, we are also involved in various activities of the parent society DGK, particularly in the organization of the 2029 IUCr Congress in Berlin. Several of our active members have been part of the organizing team since 2017; in addition, the YC teams have been vigorously promoting the event at various conferences since 2021. Finally, we successfully represented the German proposal at the 2023 IUCr General Assembly (together with the chief organizer, Dr Manfred Weiss) and have thus become an essential part of the promotional process. We will continue to devote our energies in the coming years to the success of the 2029 IUCr Congress.

Thanks to the motivated team of founders, the social network grew from 50 members at the kick-off meeting in 2013 to over 250 members from 15 different countries in 2023, leading to considerable synergy, as shown by joint publications. For instance, groups from TU Bergakademie Freiberg and TU Berlin collaborated to investigate the relative contributions of F^−^ and Li^+^ ions to the electrical conductivity of the ion conductor O:BaLiF_3_ by neutron diffraction and bond-valence site-energy (BVSE) calculations (Wiedemann *et al.*, 2018 ▸). Separately, the Salimi group at Ferdowsi University of Mashhad, Iran joined forces with the Englert group at RWTH Aachen, Germany to study the structural, environmental, and gas-adsorption properties of three Zn/Cd-based metal–organic frameworks [MOFs; FUM-153(Zn-*L*(O)H), FUM-167(Cd-*L*(O)H), FUM-176(Cd-*L*(O)NH_2_)], with the MOFs prepared by the Iranian partners and the German partners performing the structure determination (Tavakoli-Quchani *et al.*, 2024*a* ▸,*b* ▸).

## 2. Current projects and activities

Today, we are a steadily growing community with over 250 registered members and a variety of annual events. Our focus is to help early-career crystallographers grow a network and exchange ideas with one another in a variety of formats, as described below.

### 2.1. Meetings

Each year, we invite young crystallographers from all around the world to a three-day-long meeting at different locations in and around Germany. Besides ‘traditional’ conference components such as scientific talks and discussions, we encourage participants to practice their presentation and communication skills by giving a short, five-minute ‘lightning talk’ accompanying their poster contributions, setting out for the audience an entertaining ‘appetizer’ for the subject of the research. In our meetings, the presenters encounter a constructive and positive environment for their contributions, interacting mostly with other young scientists rather than the usual experienced – and sometimes demanding! – conference audience.

In addition to lightning talks, we always try to include hands-on experiences for our attendees. For example, this year’s 10th anniversary meeting held at the Deutsches Elektronen-Synchrotron (DESY) in Hamburg, Germany, included five different hands-on experiments at different beamlines of the PETRA III synchrotron. These experiments were complemented by fantastic scientific talks presented by Tobias Beck and Donatella Loru.

We are also proud that only one week prior, in September 2023, the first joint French–German young crystallographers meeting was held in Strasbourg, France. This was the kick-off meeting for the French young crystallographers, who followed our role model for the rejuvenation of crystallography in France. Both sides are enthusiastic to keep up the relationships by organizing future joint meetings.

### 2.2. Blog articles

We have established an open-access catalog of several contributions to different topics in the form of short and condensed blog articles on the website of the DGK (German Society for Crystallography, 2024 ▸; Nentwich *et al.*, 2022*a* ▸), designed for educational and informative purposes. This blog can be divided into four categories.

*Meet the People* is our format for introducing people associated with young crystallographers, such as award winners or the current chairs of the YC. We have established this section to highlight the people behind the scenes and to provide a point of contact for those interested in joining the group.

*Conference Reports* are entertaining, short, and personal summaries of either our meetings or the conferences we have attended. These reports are not necessarily written by active YC members, but also by interested attendees of our meetings or symposia.

The last of our actively maintained blog categories is *Educational Content*. These articles comprise introductions or overviews of crystallography-related topics (*e.g.*, NMR crystallography or refinement software), technical guides (*e.g.*, to useful LaTeX packages), and academic educational content (*e.g.*, on proposal or paper writing).

Thankfully, our *One Year of Pandemic* category could be discontinued. Besides other contributions, we introduced our readers to the ‘Folding@home’ initiative and suggested how to contribute to the fight against SARS-COV-19.

### 2.3. Communication: Newsletter and social media

Besides the chair’s responsibilities, there are a lot of organizational tasks in and around the YC that every member can contribute to. For example, there is a position that manages the blog posts and several social media accounts where we actively post event notices, educational content, or sometimes just humorous snippets. Our most important tool for outward communication is, however, our monthly newsletter, in which we summarize past events and notify readers of upcoming events or open positions.

Aside from this, we actively follow fellow researchers with the YC social media accounts and use these platforms to share, discuss, and highlight the work of our colleagues.

### 2.4. Lieselotte Templeton Prize

We established the Lieselotte Templeton Prize to recognize young scientists for their outstanding Bachelors or Masters (or equivalent) theses in the interdisciplinary field of crystallography. The purpose of establishing this prize was to improve the public perception of crystallography, increase the visibility of the DGK, and, above all, actively contribute to the promotion of young scientists.

Besides an award certificate, prize winners receive a travel grant to the next annual DGK conference, the opportunity to present their research at this conference, and a three-year membership in the DGK. In this way, we hope to strengthen their ties to the society in the long term.

The award is given in honor of Lieselotte Templeton who, although born in Germany, was a successful crystallographer in Berkeley, USA, and contributed enormously to the fields of resonant scattering and X-ray dichroism (Templeton & Templeton, 1982 ▸). Last but not least, the prize was named after her because she is remembered as an enthusiastic and inspiring mentor for her students. We were happy to note that the University of California, Berkeley prominently acknowledged the prize with a public statement on its website (UC Berkeley College of Chemistry, 2023 ▸).

### 2.5. Special issue ‘Spotlight on Germany’s Young Crystallographers’

Finally, possibly our biggest project so far was our special issue, which appeared in 2022 in the journal *Zeitschrift für Kristallographie*. This issue featured contributions from young crystallographers from all over Germany on a wide variety of topics, including *Theory* (Subramaniam *et al.*, 2022 ▸; Ernst *et al.*, 2022 ▸; Nentwich *et al.*, 2022*b* ▸; Hornfeck, 2022 ▸; Wiedemann, 2022 ▸), *Crystal Growth* (van Terwingen *et al.*, 2022 ▸; Weigel *et al.*, 2022 ▸, Buyer *et al.*, 2022 ▸), *Structure Solution* (Heinen *et al.*, 2022 ▸; Folkers *et al.*, 2022 ▸; Heidecker *et al.*, 2022 ▸), and *Properties* (Ludt & Zschornak, 2022 ▸; Breternitz, 2022 ▸).

**Figure 2:**
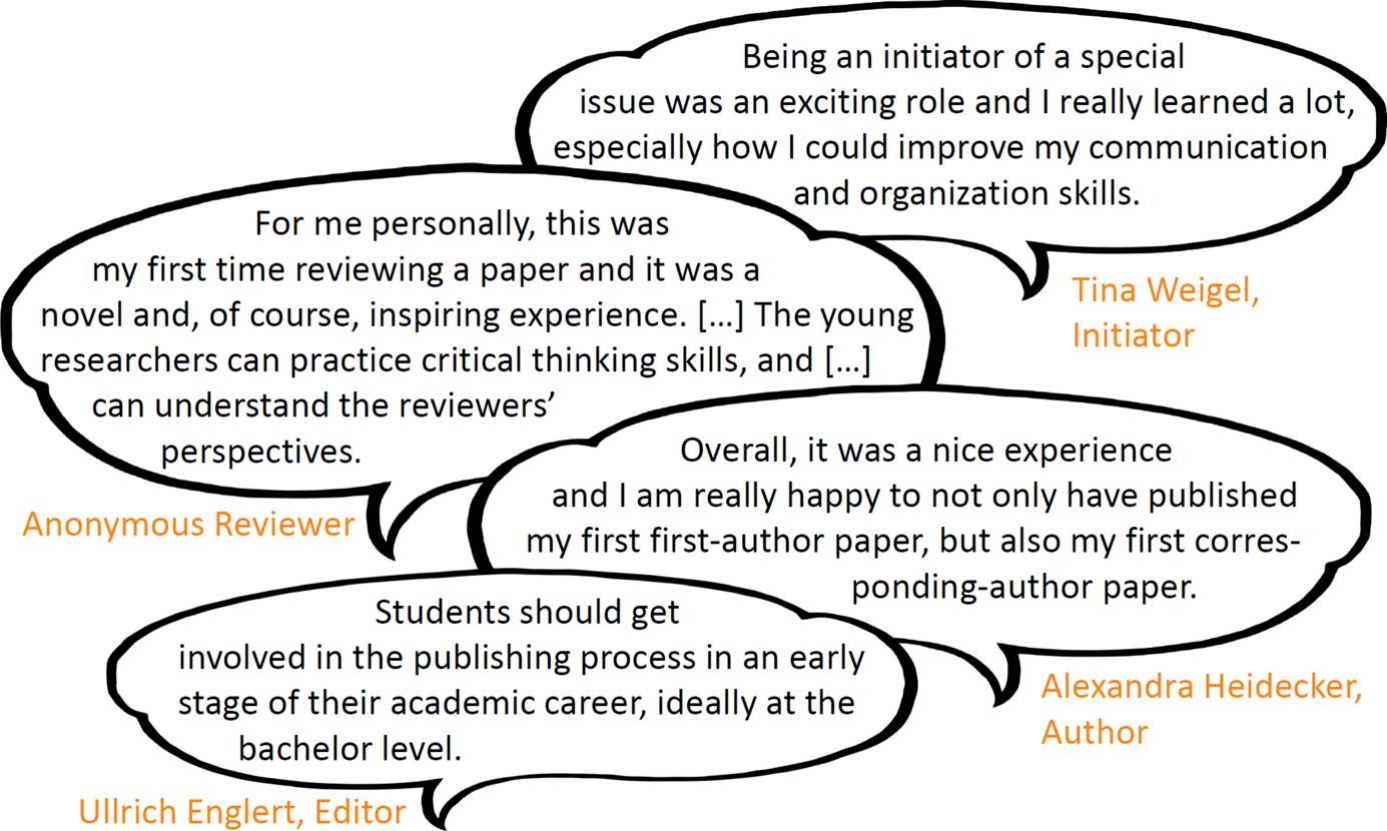
Selected comments on the special issue ‘Spotlight on Germany’s Young Crystallographers’ (Nentwich, 2022 ▸).

This issue aimed to provide an opportunity for young crystallographers to publish their work peer-reviewed in a friendly and supportive environment, as this is not always the case in normal peer review. The feedback for this special issue (see Fig. 2 ▸) from all kinds of contributors and readers has been entirely positive. We see a major gain in knowledge and experience in all aspects of publication: from initiation and organization to writing and reviewing. In order to offer this opportunity to a new crop of young scientists, we have already started working on a new spotlight special issue.

## 3. Future plans and conclusion

In the future, we want to keep and further establish our existing initiatives while exploring new possibilities. The fruitful collaborations between the French Young Crystallographers and German Young Crystal Growers over the last few years have shown us how important and inspiring joint meetings can be. The public dissemination of the details of our internal meetings, for example at DESY, is also something we will certainly continue.

Building on the success of the first published special issue, we are currently planning a second issue. As social media are the fastest and most efficient way to reach especially younger people, we will focus on communicating our events, achievements, and activities in that way.

We see the value in networking and practicing communication skills in front of a friendly audience for our members and all who attend our events.

Currently, we are also exploring the possibilities of establishing a – maybe recurring – science slam event with a focus on crystallography. It is now more than ever an opportune moment to get involved with young crystallographers. Stay tuned to our plans!
